# Functional Divergence Analysis of *AGL6* Genes in *Prunus mume*

**DOI:** 10.3390/plants12010158

**Published:** 2022-12-29

**Authors:** Lei Wang, Jinhai Song, Xu Han, Yunyan Yu, Qikui Wu, Shuai Qi, Zongda Xu

**Affiliations:** Shandong Provincial Research Center of Demonstration Engineering Technology for Urban and Rural Landscape, College of Forestry, Shandong Agricultural University, Taian 271018, China

**Keywords:** *Prunus mume*, *AGL6*, MADS-box gene, flower development

## Abstract

The *AGAMOUS-LIKE6 (AGL6)* lineage is an important clade of MADS-box transcription factors that play essential roles in floral organ development. The genome of *Prunus mume* contains two homoeologous *AGL6* genes that are replicated as gene fragments. In this study, two *AGL6* homologs, *PmAGL6-1* and *PmAGL6-2*, were cloned from *P. mume* and then functionally characterized. Sequence alignment and phylogenetic analyses grouped both genes into the *AGL6* lineage. The expression patterns and protein–protein interaction patterns showed significant differences between the two genes. However, the ectopic expression of the two genes in *Arabidopsis thaliana* resulted in similar phenotypes, including the promotion of flowering, alteration of floral organ structure, participation in the formation of the floral meristem and promotion of pod bending. Therefore, gene duplication has led to some functional divergence of *PmAGL6-1* and *PmAGL6-2* but their functions are similar. We thus speculated that *AGL6* genes play a crucial role in flower development in *P. mume*.

## 1. Introduction

*Prunus mume*, the flowers of which occupy the top position among the ten most famous flowers in China, has a planting history of more than 3000 years. The types of its flowers have changed considerably due to its long cultivation. On the basis of the study of flower development in model plants, a series of molecular regulation models for floral organ development have previously been proposed. However, the molecular mechanism regulating flower organ development in *P. mume* is largely unknown.

According to the ABCDE model, the genes that are responsible for the identity of the five floral organs are divided into five classes: A, B, C, D and E (A for the sepals; A, B and E for the petals; B, C and E for the stamens; C and E for the carpels; and C, D and E for the ovules) [1]. In *Arabidopsis*, the A-class genes are *APETALA1* (*AP1*) and *APETALA2 (AP2)*, the B-class genes are *APETALA3* (*AP3*) and *PISTILLATA* (*PI*), the C-class gene is *AGAMOUS* (*AG*), the D-class genes are *SHATTERPROOF 1* and *2* (*SHP1,2*) and *SEEDSTICK* (*STK*) and the E-class genes are *SEP1*, *SEP2*, *SEP3* and *SEP4*. According to the floral quartet model, the ABCDE class genes regulate the development of different floral organs by forming floral organ-specific tetrameric complexes [2]. In addition to the A, B, C, D and E genes, other MADS-box genes, such as *AGL6,* play important regulatory roles in plant flower development.

In contrast to the A, B, C, D and E genes, which have been widely studied, there are still few comprehensive studies on the function of the *AGL6* genes. *AGL6* genes have different expression patterns in different types of plants. In gymnosperms, *AGL6* genes are expressed in both vegetative and reproductive organs and are highly expressed in male and female structures. In *Picea abies*, *DAL1* expression is readily detected in male and female cones and in vegetative shoots [3]. In *Cryptomeria japonica*, *CjMADS14* is expressed mainly in the strobili [4]. In dicotyledonous plants, the *AGL6* genes are mainly expressed in floral organs, and some are also expressed in vegetative organs. There are two *AGL6* genes, *AGL6* and *AGL13*, in the classical model plant *Arabidopsis*. *AGL6* is expressed in ovules, while *AGL13* is specifically expressed in pollen and ovules [5,6]. *MdMADS11* mRNA expression is high in sepals, petals and ovaries with receptacles [7]. In *Actinidia chinensis*, two *AGL6* homologues have obtained different expression patterns: *AcAGL6a* is expressed in flower parts and all vegetative tissue samples but mainly in vegetative branches, while *AcAGL6b* is expressed in vegetative and floral tissues (sepals and petals) but mainly in flower tissues [8]. In monocots, the *AGL6* gene is mainly expressed in floral organs. *PaAGL6-1* and *PaAGL6-2* are expressed throughout all floral organs in *Phalaenopsis aphrodite*, and *PaAGL6-1* is highly expressed specifically in the lip [9]. In *Alpinia hainanensis*, *AhAGL6*-like is expressed in all six mature floral organs [10]. Although the expression patterns of *AGL6* genes in angiosperms and gymnosperms are slightly different, they are both expressed in floral organs, indicating that *AGL6* genes are involved in the development of floral organs. Although *AGL6* genes are expressed in the flowers of different plants, there are obvious differences in their expression patterns. *PhAGL6* from *P. hybrida* is present at high levels in developing petals and ovaries, *CpAGL6* from *Chimonanthus praecox* is highly expressed in middle-tepal tissue and *HoAGL6* from *Hyacinthus orientalis* is highly expressed in ovules [11,12,13]. This indicates that, while *AGL6* genes may be involved in the regulation of floral organ development in different plants, their functions have undergone species-specific differentiation.

The analysis of *AGL6* gene mutants or plants overexpressing the *AGL6* gene also proved that the *AGL6* gene is involved in floral organ regulation. The lodicules, stamens and carpels were all found to be absent in an *OsMADS6* mutant, and many green and lemma-like organs appeared in their place. However, overexpression of *OsMADS6* results in the overproduction of palea, lodicules, stamens and carpels [14]. *bde* mutants exhibit multiple defects in floral development, but these defects do not appear during earlier stages of inflorescence or vegetative development [15]. In *Arabidopsis*, the flowering time and fertility of *agl6-2*-deletion mutant plants are normal, which may be due to the redundant function of another *AGL6* gene, *AGL13*. *Arabidopsis* plants overexpressing *AGL6* produce terminal flowers at the top of the inflorescence, and the number and morphology of the floral organs of the terminal flowers are altered [5], suggesting that *AGL6* regulates the differentiation of floral organs in *Arabidopsis*. The ectopic expression of *OMADS1*, an orchid (*Oncidium* Gower Ramsey) *AGL6*-like gene, results in the production of terminal flowers in transgenic *Arabidopsis* plants [16].

In addition to regulating the development of floral organs, *AGL6* genes also regulate flowering time: *AGL6* can shorten the growth period of young plants and promote early flowering. *Arabidopsis* plants overexpressing the *AGL6* gene show an early-flowering phenotype under both long-day and short-day conditions [17]. *Arabidopsis* plants overexpressing the *AGL6* genes of different plant species, such as wintersweet and hyacinth, show shorter vegetative growth times and earlier flowering times [11,13]. The *AGL6* gene *DAL1* plays an important regulatory role in the juvenile-to-adult phase transition in *Norway spruce*. *Arabidopsis* plants transformed with the *DAL1* gene show an early-flowering phenotype, and plants with an extremely early-flowering phenotype bud and flower as soon as the cotyledons are unearthed [3]. The above results suggest that promoting the expression of the *AGL6* gene is a feasible approach for shortening the juvenile growth period and promoting early flowering of plants, especially woody plants.

Gene duplication plays an important role in the evolution of diversity and novel functions and is especially prevalent in the nuclear genomes of flowering plants [18]. Duplicated genes (paralogs) may experience the following fates: (1) nonfunctionalization through silencing or null mutation, (2) neofunctionalization through the acquisition of a novel function or (3) subfunctionalization through the partitioning of functional modules such that the complementation of the two copies provides the functional capability of the ancestral gene [18]. Extensive duplication events have resulted in the expansion of MADS-box genes, and the study of MADS-box gene evolution provides examples of all three postduplication fates mentioned above [19,20].

According to the existing research, there is more than one *AGL6* gene within individual species. There is only one *AGL6* gene in *Brachypodium distachyon*, ginger and petunia [12,21,22,23,24], while two *AGL6*-type genes are found in *Arabidopsis*, *Magnolia wufengensis* and rice [6,17,25,26,27] and three *AGL6*-class genes are present in wheat and *Cymbidium goeringii* [28,29]. In *Rosa chinensis*, the number of *AGL6* genes reaches five [30]. The current research on *AGL6* genes is not sufficient, and more *AGL6* genes remain to be found.

The two *AGL6* genes of *P. mume* are replicated as gene fragments and, therefore, provide a good model for studying the functional divergence and evolution of duplicated genes in plants. *AGL6* genes do not belong to any of the ABDCE gene categories, but they play important roles in flower development and floral organ determination in plants. In this study, we cloned two *AGL6* homologs, *PmAGL6-1* and *PmAGL6-2*, from *P. mume*. We studied their phylogeny, expression patterns and protein–protein interaction patterns and analyzed their functions. On this basis, the model of the molecular regulation of *P. mume* organ development was improved to provide a theoretical basis for using the *AGL6* gene to improve the flower types of *P. mume*.

## 2. Results

### 2.1. Cloning and Sequence Analysis of PmAGL6-1 and PmAGL6-2

**Figure 1:**
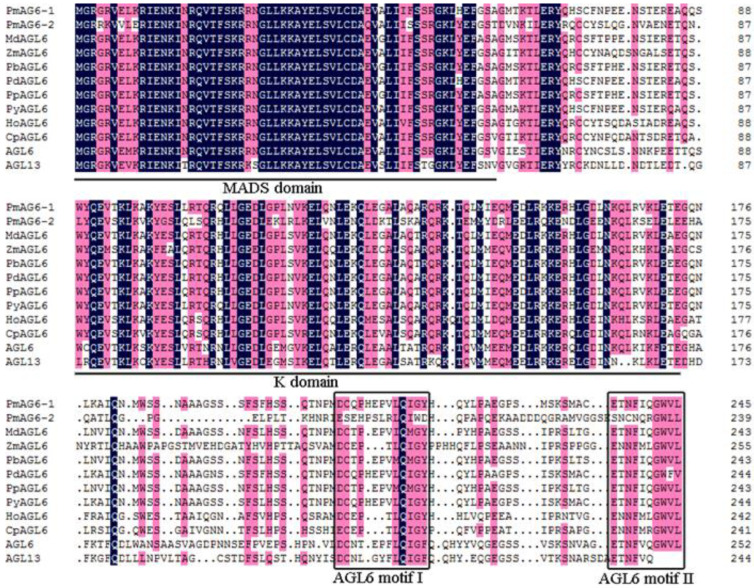
Sequence alignment of *PmAGL6-1*, *PmAGL6-2* and *AGL6* proteins from other plants. The MADS domain and K domain regions are underlined, and *AGL6* motifs I and II are boxed. Accession numbers for the aligned sequences are as follows: MdAGL6 (*Malus domestica*, NP_001280892), ZmAGL6 (*Zea mays*, NP_001105332.1), PbAGL6 (*Pyrus×bretschneideri*, XP_009375842.1), PdAGL6 (*Prunus dulcis*, BBG97451), PpAGL6 (*Pyrus pyrifolia* var. *culta,* BAK18784.2), PyAGL6 (*Prunus yedoensis* var. *nudiflora*, PQQ17269.1), HoAGL6 (*Hyacinthus orientalis*, AAT88088.1), CpAGL6 (*Chimonanthus praecox*, ACN88212.1), *AGL6* (*Arabidopsis thaliana*, NP_182089) and AGL13 (*Arabidopsis thaliana*, NP_191671).

**Figure 2:**
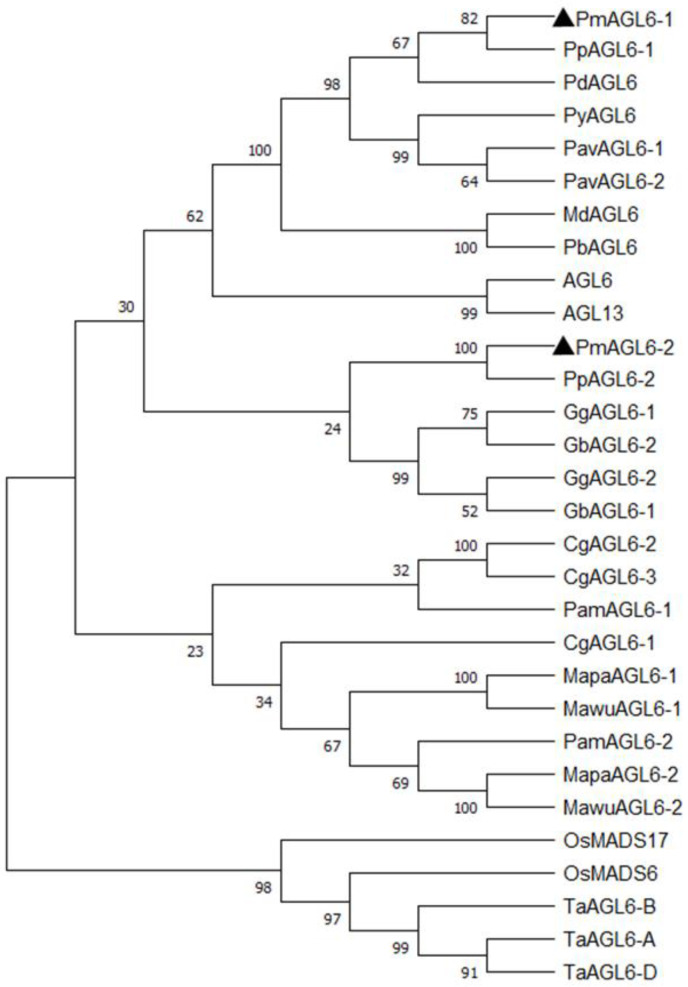
Phylogenetic analysis of AGL6 proteins. The tree was constructed via the neighbor-joining method using MEGA5-X software with a bootstrap analysis of 1000 replicates. The accession numbers of the other proteins included in the phylogenetic analysis are as follows: PpAGL6-1 (*Prunus persica*, XP_020412713.1), PpAGL6-2 (*Prunus persica*, XP_007220094), PdAGL6 (*Prunus dulcis*, BBG97451), MdAGL6 (*Malus domestica*, NP_001280892), PavAGL6-1 (*Prunus avium*, XP_021825726.1), PavAGL6-2 (Prunus avium, XP_021825734.1), PyAGL6 (*Prunus yedoensis* var. *nudiflora*, PQQ17269.1), PbAGL6 (*Pyrus×bretschneideri*, XP_009375842.1), PamAGL6-1 (*Persea Americana*, ABG49493.1), PamAGL6-2 (Persea Americana, ABG49494.1), MapaAGL6-1 (*Magnolia patungensis*, ATB53133.1), MapaAGL6-2 (*Magnolia patungensis*, ATB53135.1), MawuAGL6-1 (*Magnolia wufengensis*, AOZ15901.1), MawuAGL6-2 (*Magnolia wufengensis*, AOZ15902.1), TaAGL6-A (*Triticum aestivum*, AYP71082.1), TaAGL6-B (*Triticum aestivum*, AYP71084.1), TaAGL6-D (*Triticum aestivum*, AYP71083.1), AGL6 (*Arabidopsis thaliana*, NP_182089), AGL13 (*Arabidopsis thaliana*, NP_191671), CgAGL6-1 (*Cymbidium goeringii*, ACT66279.1), CgAGL6-2 (*Cymbidium goeringii*, APY18455.1), CgAGL6-3 (*Cymbidium goeringii*, AMW17745.1), OsMADS6 (*Oryza sativa*, Q6EU39.1), OsMADS17 (*Oryza sativa*, ACX35552.1), GgAGL6-1 (*Gnetum gnemon*, CAB44455.1), GgAGL6-2 (*Gnetum gnemon*, CAB44457.1), GbAGL6-1 (*Ginkgo biloba*, BAD93172.1) and GbAGL6-2 (*Ginkgo biloba*, AIC79629.1).

The full-length CDSs of *PmAGL6-1* and *PmAGL6-2* were cloned and then confirmed by sequencing (Figure 1). *PmAGL6-1* contained a 738 bp open reading frame encoding 245 amino acids, whereas *PmAGL6-2* contained a 720 bp open reading frame encoding 239 amino acids. The two genes also included a highly conserved MADS domain and a less conserved K domain (Figure 1). In addition, *AGL6* motifs I and II [26] were identified in the C-terminal domains of *PmAGL6-1* and *PmAGL6-2*. The phylogenetic analysis of *AGL6* proteins showed that *PmAGL6-1* and *PmAGL6-2* belonged to two separate subclades (Figure 2).

### 2.2. Expression Pattern Analysis of PmAGL6-1 and PmAGL6-2

**Figure 3:**
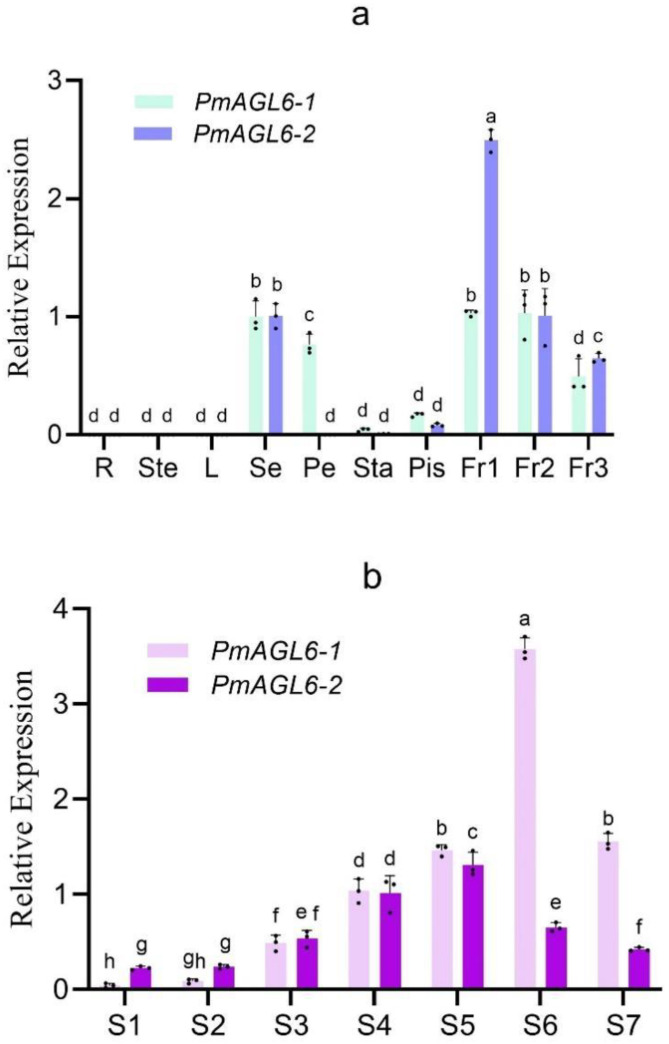
Expression patterns of *PmAGL6-1* and *PmAGL6-2*. (**a**) Expression patterns of *PmAGL6-1* and *PmAGL6-2* in different organs of *P. mume*. R: root, Ste: stem, L: leaf, Se: sepal, Pe: petal, Sta: stamen, Pis: pistile, Fr1–3: fruit development stages 1–3. (**b**) Expression patterns of *PmAGL6-1* and *PmAGL6-2* during *P. mume* floral bud differentiation. S1: flower primordium formation, S2: sepal initiation, S3: petal initiation, S4: stamen initiation, S5: pistil initiation, S6: ovule development, S7: anther development. Significant differences are identified by SPSS with Duncan’s test (*p* < 0.05) and are represented by different letters above the error bars.

The expression patterns of *PmAGL6-1* and *PmAGL6-2* were analyzed by qRT-PCR (Table 1). The transcripts of *PmAGL6-1* and *PmAGL6-2* were not detected in the vegetative organs (roots, stems and leaves), and the expression domains of the two genes were limited to reproductive organs. The tissue-specific expression of these two genes in floral tissues has diverged. The *PmAGL6-1* gene is expressed in sepals, petals, stamens and pistils, but its expression levels are very different. The expression levels are the highest in sepals and petal and low in pistils and stamens. The *PmAGL6-2* gene is expressed at the highest level in sepals and at a low level in pistils. *PmAGL6-1* and *PmAGL6-2* were all highly expressed in the three fruit stages and the expression level decreased gradually with the development of fruit (Figure 3a).

During the differentiation of flower buds, the expression levels of *PmAGL6-1* and *PmAGL6-2* first increased until they reached a peak and then decreased. *PmAGL6-1* reached its highest expression level in S6, while *PmAGL6-2* was expressed at the highest level in S5 (Figure 3b).

### 2.3. Ectopic Expression of PmAGL6-1 and PmAGL6-2 in Arabidopsis

**Figure 4:**
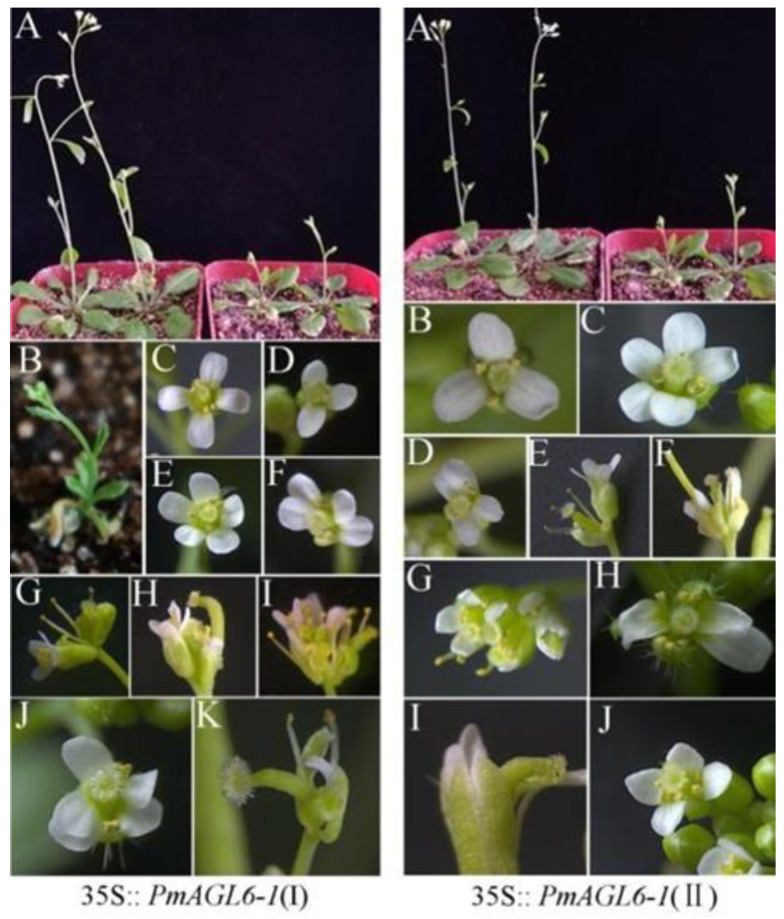
Functional analysis of *PmAGL6-1* and *PmAGL6-2* by transforming *Arabidopsis* plants. (**IA**–**IK**) Constitutive expression in 35S::*PmAGL6-1* transgenic *Arabidopsis* plants. (**IIA**–**IIJ**) Constitutive expression in 35S:: *PmAGL6-2* transgenic *Arabidopsis* plants. (**IA**) Early-flowering phenotype (A4 line) following the ectopic expression of *PmAGL6-1* (left) and normal vegetative growth in the wild-type (right). (**IIA**) Early-flowering phenotype (B4 line) following the ectopic expression of *PmAGL6-2* (left) and normal vegetative growth in the wild-type (right). (**IB**) Extremely early-flowering phenotype (A1 line) following the ectopic expression of *PmAGL6-1*. (**IC**) Flower from wild-type *Arabidopsis*. (**ID**,**IIB**) Flower with three petals. (**IE**,**IIC**) Flower with five petals. (**IF**,**IID**) Bilaterally symmetrical flower. (**IG**,**IIE**) The top two flowers of the inflorescence combine to form a single flower. (**IH**,**IIF**) The top three flowers of the inflorescence combine to form a single flower. (**II**,**IIG**) The top four flowers of the inflorescence combine to form a single flower. (**IJ**,**IIH**) Flower with overlapping petals. (**IK**,**III**) Curved fruit pod. (**IIJ**) Flower with a sharp petal apex.

The seeds of the T2-generation transgenic plants of different lines were screened and sown with wild-type *Arabidopsis* at the same time. The phenotypes of T2-generation transgenic plants obtained under the same environmental conditions were observed. The ectopic expression of *PmAGL6-1* and *PmAGL6-2* caused early flowering (Figure 4IA,IIA and Table 2). Extremely early flowering was observed in *PmAGL6-1* transgenic plants, which showed curling leaves and shorter plant heights (Figure 4IB) than wild-type *Arabidopsis* plants. In addition to the changes in flowering time, petal numbers and flower architecture were also changed in the transgenic plants. The number of petals in the transgenic plants varied from three to five (Figure 4ID,IE,IIB,IIC). The overexpression of *PmAGL6-1* and *PmAGL6-2* resulted in bilaterally symmetrical petals in transgenic *Arabidopsis* plants (Figure 4IF,IID) and changed the arrangement of the petals, resulting in the partial overlap of petals (Figure 4IJ,IIH). In addition, in *PmAGL6-2* transgenic plants, the petal tip became pointed (Figure 4IIJ). Terminal flowers were observed in *PmAGL6-1* and *PmAGL6-2* transgenic plants in which two to four flowers were combined (Figure 4IG–II,IIE–IIG). Both types of transgenic plants exhibited curved fruit pods (Figure 4IK,III).

**Figure 5:**
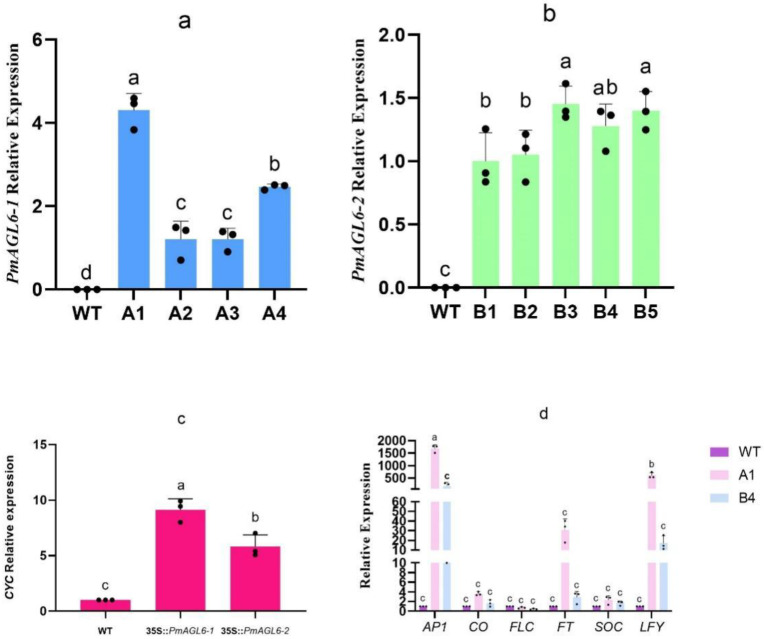
Analysis of related gene expression in transformants. (**a**) Expression of *PmAGL6-1* in five transgenic Arabidopsis plants. (**b**) Expression of *PmAGL6-2* in five transgenic Arabidopsis plants. (**c**) Expression of *CYC* in transgenic plants. (**d**) Expression of flowering regulators in the 35S::*PmAGL6-1* transformant A1 line and the 35S::*PmAGL6-2* transformant B4 line. Significant differences are identified by SPSS with Duncan’s test (*p* < 0.05) and are represented by different letters above the error bars.

We analyzed the expression profiles of *PmAGL6-1* in four 35S::*PmAGL6-1* transgenic *Arabidopsis* lines and *PmAGL6-2* in five 35S::*PmAGL6-2* transgenic *Arabidopsis* lines by qRT-PCR. The results showed that *PmAGL6-1* and *PmAGL6-2* were expressed in the transgenic plants, but their expression levels were different in different lines (Figure 5a,b).

*CYC* genes play an important role in flower symmetry [31,32]. To further understand the reasons why the flowers became bilaterally symmetrical in the *PmAGL6-1* and *PmAGL6-2* transgenic *Arabidopsis* plants, we collected bilaterally symmetrical flowers from 35S::*PmAGL6-1* and 35S::*PmAGL6-1* transgenic *Arabidopsis* plants. Then, the expression of the *CYC* gene was analyzed. In bilaterally symmetrical flowers from the 35S::*PmAGL6-1* and 35S::*PmAGL6-2* transgenic *Arabidopsis* plants, *CYC* was upregulated (Figure 5c).

To further understand the mechanisms responsible for the early flowering of the *PmAGL6-1* and *PmAGL6-2* transgenic *Arabidopsis* lines, the expressions of some of the endogenous flowering-related genes were analyzed in the 35S::*PmAGL6-1* transformant A1 line and the 35S::*PmAGL6-2* transformant B4 line, which exhibited an early-flowering phenotype. Relative to the wild-type, *AP1*, *CO*, *FLC*, *FT*, *SOC* and *LFY* expression was upregulated in the two early-flowering lines, whereas the expression of *FLC* was downregulated (Figure 5d).

### 2.4. Protein–Protein Interactions among the Products of Two AGL6 Genes and Other Floral Organ Identity-Determining Genes in P. mume

**Figure 6:**
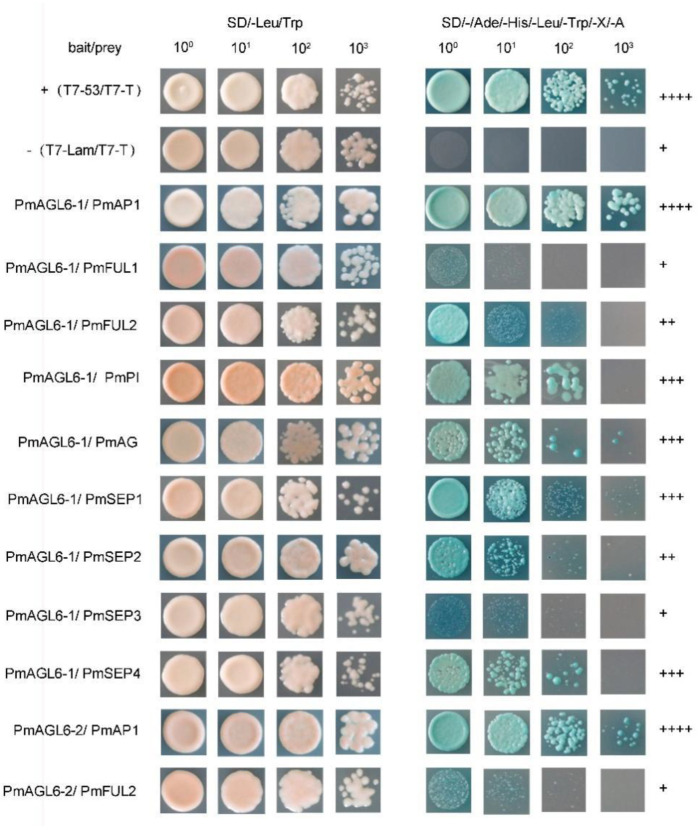

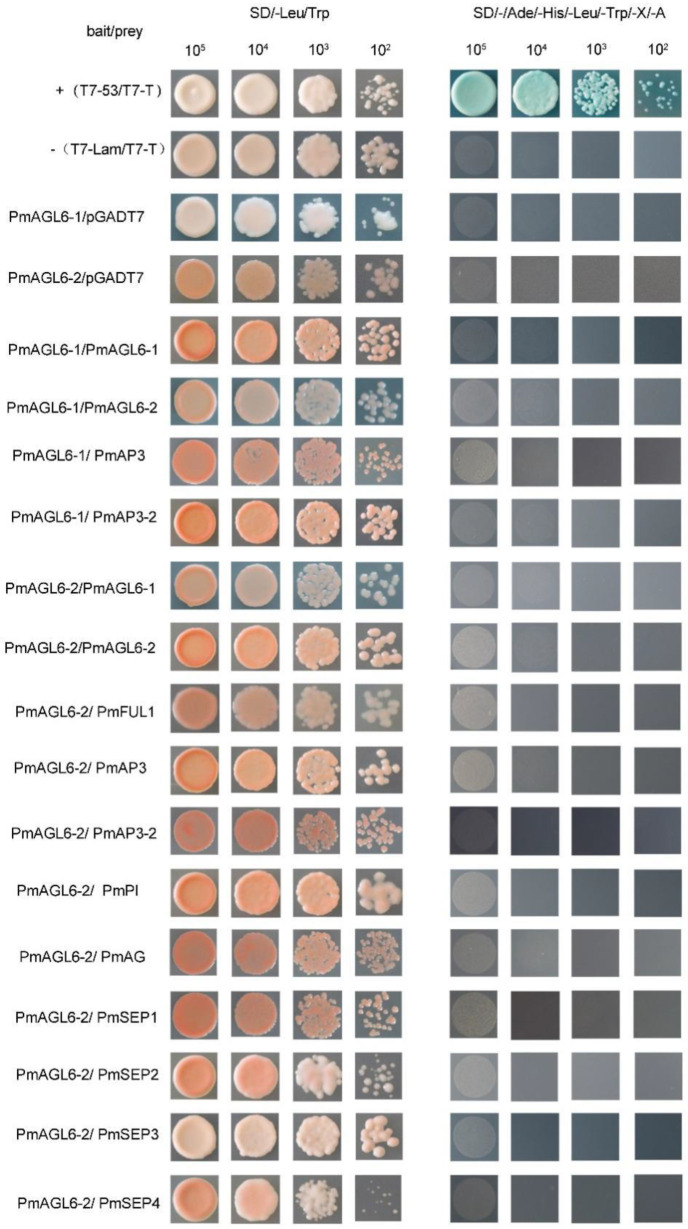
Positive results of the Y2H analysis of protein–protein interactions among the products of the two *AGL6* genes and other floral organ identity-determining genes in *P. mume*. T7-53/T7-T and T7-Lam/T7-T were the positive and negative controls, respectively. + represents the intensity of the interaction and − indicates no interaction.

We used yeast two-hybrid assays and bimolecular fluorescence complementation experiments to study protein–protein interactions among the products of two *AGL6* genes and other floral organ identity-determining genes in *P. mume*. The Y2H experiment revealed that neither *PmAGL6-1* nor *PmAGL6-2* could form a homodimer and that they could not interact with each other. There were differences in the types and numbers of interacting proteins between the products of the two *AGL6* proteins. Nine proteins interacted with *PmAGL6-1* among the A, B, C and E gene products, while only two class A genes showed interactions with *PmAGL6-2*. *PmAGL6-1* could strongly dimerize with PmAP1 and moderately interacted with PmFUL2, PmPI, PmAG, PmSEP1, PmSEP2 and PmSEP4. *PmAGL6-1* could only interact weakly with PmFUL1 and PmSEP3. *PmAGL6-2* also showed strong interactions with PmAP1 but weaker interactions with PmFUL2 (Figure 6).

**Figure 7:**
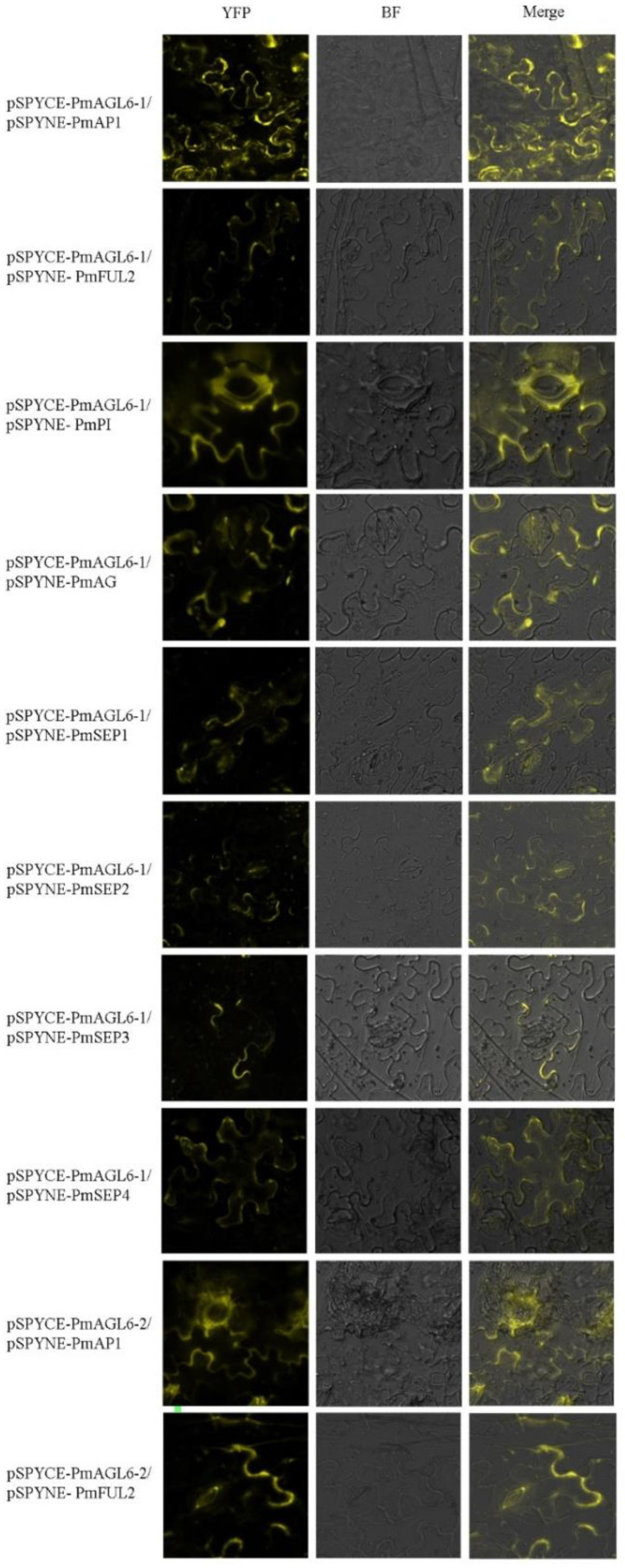

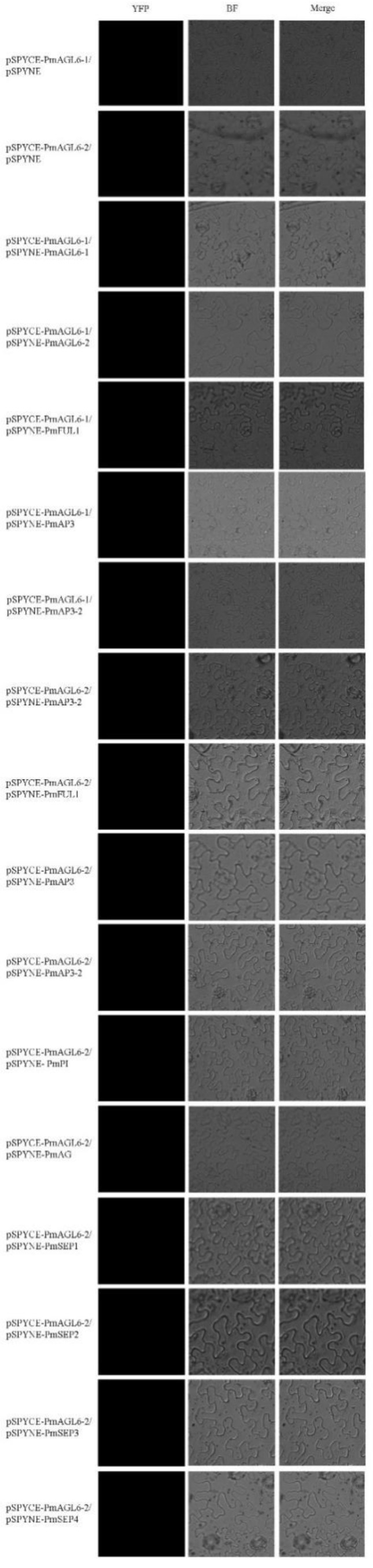
Positive results of the BiFC analysis of protein–protein interactions among the products of the two *AGL6* genes and other floral organ identity-determining genes in *P. mume*. YFP: yellow fluorescent protein, BF: bright-field image, merged: merged YFP and BF images.

To further verify the results of the yeast two-hybrid assays, we carried out fluorescence complementation experiments. The results obtained via the two experimental methods were basically the same. Yellow fluorescent protein (YFP) signals were detected in the tobacco epidermis cell membrane when the tested combination showed interaction in the Y2H analysis, except for YCE-*PmAGL6-1*/YNE-PmFUL1, possibly because the interaction between *PmAGL6-1* and PmFUL1 was too weak or nonexistent (Figure 7).

## 3. Discussion

In this study, two *AGL6* genes, *PmAGL6-1* and *PmAGL6-2*, from *P. mume* were cloned and analyzed. The open reading frame of *PmAGL6-2* was 18 bases shorter than that of *PmAGL6-1*, and the missing bases were located mainly in the C region. *PmAGL6-1* shared 51.02% homology with *PmAGL6-2*. The amino acid sequences of the K region, AGL6-I motif and AGL6-II motif of the two genes were very different. Phylogenetic tree analysis also showed that the two genes were not clustered together. This suggested that the sequences of the two genes diverged after the duplication events.

*PmAGL6-1* and *PmAGL6-2* were both highly expressed in fruit, indicating that the two *AGL6* genes may be involved in the development of fruits in *P. mume*. Neither *PmAGL6-1* nor *PmAGL6-2* was expressed in vegetative tissues, and their expressions patterns in floral organs were also different. *PmAGL6-1* was expressed in sepals, petals, stamens and pistils, and it was highly expressed in sepals and petals. *PmAGL6-2* was only expressed in sepals and carpels and was highly expressed in sepals. This expression pattern in floral organs is similar to *PmAP1* in *P. mume* [33]. The expression patterns of *PmAGL6-1* and *PmAGL6-2* in different flower bud differentiation stages were the same, first increasing and then decreasing after they reached a maximum. This expression pattern is consistent with those of the *AGL6* genes of wheat and wintersweet [13,28] and also similar to the expression pattern of *PmAP1* in *P. mume* [33]. It can be seen that *PmAGL6-1* and *PmAGL6-2* are involved in the development of floral organs in *P. mume*, and their expression patterns are similar to the expression pattern of *PmAP1*.

*PmAGL6-1* can interact with the class A, B, C and E genes in *P. mume*, while *PmAGL6-2* only interacts with class A genes. In wheat, three TaAGL6 proteins interact with TaAP3, TaAG and TaMADS13, and redundant functions for these proteins have been suggested [28]. Both MawuAGL6-1 and MawuAGL6-2 show strong interactions with MawuAGL2, MawuAGL9 and MawuAG-1 and can form homodimers and interact with each other; one difference is that MawuAGL6-2 can interact with MawuAP1 [25], and their protein–protein interaction patterns differ slightly. In contrast, the protein–protein interaction patterns of *PmAGL6-1* and *PmAGL6-2* are very different. Studies have shown that the K region of the MADS-box gene plays a significant role in protein–protein interactions [34,35]. The amino acid sequences of the K regions of the three *AGL6* genes in wheat are identical, which may cause them to interact with the same proteins. There are 14 amino acid differences in the K region of the amino acid sequence encoded by *MawuAGL6-1* and *MawuAGL6-2*, and 28 amino acid differences in the K region of the amino acid sequence encoded by *PmAGL6-1* and *PmAGL6-2*; this may be the fundamental reason why the difference in the protein–protein interaction patterns between *PmAGL6-1* and *PmAGL6-2* was greater than that between MawuAGL6-1 and MawuAGL6-2. The similarity in the protein–protein interaction patterns between *PmAGL6-1* and *PmAGL6-2* was that they could interact with PmAP1 and PmFUL2. The protein–protein interaction patterns of *PmAGL6-1* and PmAP1 were very similar, further implying the similarity of their functions [33].

The overexpression of *PmAGL6-1* and *PmAGL6-2* resulted in early-flowering phenotypes in *Arabidopsis*. Moreover, a very early-flowering phenotype was found in *PmAGL6-1* transgenic plants, which was very similar to the very early-flowering phenotype caused by the ectopic expression of *OMADS7*, *OMADS1* and *DAL* in *Arabidopsis* in terms of plant appearance [3,36]. *OMADS1* significantly promotes flowering by positively activating the flowering time genes *FT* and *SOC1* and the floral initiation genes *LFY* and *AP1* in transgenic *Arabidopsis* plants [16]. Upregulated expression of *HoAGL6* causes early flowering by increasing the expression of *SOC1* and *LFY* [11]. The ectopic expression of *CpAGL6* in *Arabidopsis* leads to precocious flowering, which is mainly correlated with the inhibition of the floral repressor *FLC* and the promotion of the floral promoters *AP1* and *FT* [13]. Endogenous overexpression of *AGL6* increases the expression of the known floral regulators *FT* and *AP1* in *Arabidopsis* [5]. *FT*, *SOC* and *CO* are flowering-time genes that are close to the top of the regulatory hierarchy in flower development and mediate the switch from vegetative to reproductive development by activating meristem identity genes or by repressing genes that maintain the vegetative phase. *AP1* and *LFY* are meristem identity genes that control the transition from vegetative growth to inflorescence and floral meristem development and function as upstream regulators of floral organ identity genes [37,38,39,40]. *FLC* is an inhibitor of flowering in *Arabidopsis* [41]. The results of the detection of flowering gene expression in transgenic plants showed that *AP1*, *CO*, *FT*, *SOC* and *LFY* were upregulated in both *PmAGL6-1* and *PmAGL6-2* transgenic plants, while *FLC* was downregulated. These genes whose expression levels were measured are the key genes in the flower induction pathway: they determine a relationship between regulation and being regulated. However, based on the current research, it is impossible to determine the mechanism used by the two *PmAGL6* genes to promote flowering. We speculate that *PmAGL6-1* and *PmAGL6-2* may promote the expression of the flowering-time genes *CO*, *FT* and *SOC*; reduce the expression of *FLC*; promote the expression of the meristem identity genes *AP1* and *LFY*; and then promote flowering. As for which genes *PmAGL6-1* and *PmAGL6-2* directly act on to cause early flowering, further research is needed.

*CYC* was upregulated in the bilaterally symmetrical flowers of 35S::*PmAGL6-1* and 35S::*PmAGL6-2* transgenic *Arabidopsis* plants. This shows that the ectopic expression of *PmAGL6-1* and *PmAGL6-2* promotes the expression of *CYC* in transgenic plants, which in turn affects the symmetry of flowers. In transgenic *Arabidopsis thaliana*, the ectopic expression of *PmAGL6-1* and *PmAGL6-2* changes the number and arrangement of *Arabidopsis* petals. In addition, some petals of *PmAGL6-2* transgenic plants were sharp at the apex. In *Arabidopsis* flowers, the initiation of the four petals depends on the size of the floral meristem, the establishment of boundaries that demarcate the position of petal primordia on the floral meristem, the transient development of auxin activity maxima at these positions and the general mechanisms of lateral organ outgrowth [38,42,43]. The ectopic expression of *PmAGL6-1* and *PmAGL6-2* may affect the expression of genes related to petal development and cause the petals of transgenic plants to become abnormal. The ectopic expression of *PmAGL6-2* makes *Arabidopsis* petals change in number, arrangement and shape, but *PmAGL6-2* is not expressed in *Prunus mume* petals, indicating that *PmAGL6-2* may not participate in the development regulation of petals, but its transfer into *Arabidopsis* will cause petal changes. *ZAG3* is expressed in floral meristems. *OMADS1* is also expressed in floral meristems, and ectopic expression makes transgenic *Arabidopsis* plants produce terminal flowers [15,16]. Terminal flowers appeared in *PmAGL6-1* and *PmAGL6-2* transgenic *Arabidopsis*, indicating that *PmAGL6-1* and *PmAGL6-2* may be involved in the regulation of floral meristems. Both *PmAGL6-1* and *PmAGL6-2* can make *Arabidopsis* fruit bend, and the two genes are highly expressed in fruit, indicating that *PmAGL6-1* and *PmAGL6-2* may participate in the development of fruit in *P. mume*.

The roles of *AGL6* genes in *Oncidium* Gower Ramsey and *Nymphaea tetragona* are considered to be similar to that of *AP1* [16,44]. The study of *PmAP1* in *P. mume* showed that *PmAP1* is expressed only in sepals; similarly, the two *AGL6* genes were also expressed in sepals. The expression pattern of *PmAP1* was similar to those of *PmAGL6-1* and *PmAGL6-2*, and both *PmAGL6-1* and *PmAGL6-2* can interact strongly with PmAP1. *PmAP1* promoted early flowering in *Arabidopsis* and transformed the inflorescence meristem into the floral meristem, which has also been observed in *PmAGL6-1* and *PmAGL6-2* transgenic *Arabidopsis* (Xu 2015). From this point of view, the functions of the two *PmAGL6* genes and *PmAP1* are similar, but the two *PmAGL6* genes have their own unique functions.

In many plants, *AGL6* genes have experienced gene duplication events, producing several *AGL6* genes, but the functional changes in *AGL6* genes are different in different species. Although the two *AGL6* genes of *P. mume* differ greatly in their gene sequences, expression patterns and protein–protein interaction patterns, they exhibit important, similar functions in promoting flowering and floral organ development, especially petal development. In *Arabidopsis*, *AGL6* and *AGL13* exhibit different expression patterns and functions, showing subfunctionalization [6,17,45,46,47]. There are two *AGL6* genes in *Magnolia wufengensis*, which arose via gene duplication and underwent diversification of their expression and protein–protein interaction patterns thereafter. Functional analysis revealed that, in addition to showing common functions in accelerating flowering, *MawuAGL6-1* might be responsible for flower meristem determinacy, while *MawuAGL6-2* is preferentially recruited to regulate tepal morphogenesis [25]. Rice contains two *AGL6*-like genes: OsMADS17 plays minor and redundant roles, whereas *OsMADS6* is a key regulator of flower development [26,27,48]. The expression of *MapaAGL6-1* and *MapaAGL6-2* differs, and the ectopic expression of *MapaAGL6-1* and *MapaAGL6-2* in *Arabidopsis ap1–10* mutants results in different phenotypes [49]. Three *TaAGL6* genes exhibit similar expression patterns and protein–protein interaction patterns and show similar functions in transgenic *Arabidopsis* [28]. *CgAGL6-1*, *CgAGL6-2* and *CgAGL6-3* present different expression patterns and protein–protein interaction patterns [29]. Thus, it can be seen that there is species specificity in the differences in the expression patterns, protein–protein interaction patterns and functions of *AGL6* genes, without following any obvious rules. Therefore, further research on the *AGL6* gene should be conducted.

*P. mume* has many floral patterns, but the molecular mechanism of floral organ development in *P. mume* is still unclear. *PmAGL6-1* and *PmAGL6-2* play important roles in promoting flowering and floral organ development. Gene duplication events cause differences in the expression patterns and protein–protein interaction patterns of *PmAGL6-1* and *PmAGL6-2*, but their functions are similar. Therefore, we believe that the two *AGL6* genes should be included in the molecular model of floral organ development in *P. mume*. The growth period of *P. mume* is long, and the flowering period is one of the important factors affecting the horticulture characters of *P. mume*. Additionally, the *AGL6* gene of *P. mume* can promote flowering. By promoting the expression of *AGL6* genes, it may be possible to shorten the growth period of *P. mume* and advance the flowering time, thus improving the ornamental value of *P. mume*. In conclusion, our research provides valuable information for the study of flower organ development in other plants and as a supplement to the floral organ development model.

## 4. Materials and Methods

### 4.1. Plant Material

All samples were collected from *P. mume* “Sanlun Yudie” growing under natural conditions in the Jiufeng International Plum Blossom Garden in Beijing, China (40°07′ N, 116°11′ E). Samples were collected, and the stages of flower bud development were determined using the method described by Zhou et al. (2019) [50]. All samples were quickly frozen in liquid nitrogen and stored at −80 °C until RNA extraction.

The *Arabidopsis* Columbia ecotype was used to produce the transgenic material. After 3 days of vernalization at 4 °C in darkness, the seeds were sown in flower pots and placed in a growth chamber at 21 °C under a 16 h light/8 h dark cycle.

The tobacco (*Nicotiana benthamiana*) plants used for bimolecular fluorescence complementation (BiFC) were raised in a greenhouse under a light/dark cycle of 16 h/8 h at 25 °C.

### 4.2. Screening of AGL6 Gene in Prunus mume

The *Prunus mume* genome data were downloaded from the *Prunus mume* genome database http://prunusmumegenome.bjfu.edu.cn/ (accessed on 5 December 2022) (Zhang, 2012). Following Xu’s work, two methods, BlastP and HMMER, were used for gene screening (2015).

### 4.3. Extraction of Total RNA and Synthesis of First-Strand cDNA

Total RNA was extracted from floral buds using an EASYspin Plant RNA Rapid Extraction Kit (Aidlab Biotech, Beijing, China) following the manufacturer’s protocol, and the qualified RNA was preserved at −80 °C. First-strand cDNA was synthesized with a 5× All-In-One RT MasterMix Reverse Transcription Kit (ABM Company, Vancouver, BC, Canada) in accordance with both the manufacturer’s protocol and the requirements of RT-PCR and qRT-PCR.

### 4.4. Cloning of the Full-Length CDSs of PmAGL6-1 and PmAGL6-2

*PmAGL6-1F*, *PmAGL6-1R, PmAGL6-2F* and *PmAGL6-2R* were designed based on CDSs annotated in the genome database. PCR was performed starting with a 60 s predenaturation step at 94 °C, followed by 30 cycles of 10 s of denaturation at 98 °C, 15 s of annealing at 60 °C and 30 s of extension at 68 °C.

### 4.5. Sequence Alignment and Phylogenetic Analysis of PmAGL6-1 and PmAGL6-2

ClustalW was used to perform multiple protein sequence alignment of *PmAGL6-1*, *PmAGL6-2* and ten *AGL6* genes from other plants. The phylogenetic tree was constructed via the neighbor-joining method using MEGA 5-X software with a bootstrap analysis of 1000 replicates.

### 4.6. Real-Time Quantitative RT-PCR

We analyzed gene expression via qRT-PCR on a Bio-Rad CFX96™ Real-Time PCR instrument (Bio-Rad, Inc., Hercules, CA, USA). PP2A was used as the reference gene in the analysis of expression patterns of *PmAGL6-1* and *PmAGL6-2*. Actin was used as the reference gene in transgenic *Arabidopsis* (Table 1). The CYC was extracted from the flower buds of transgenic *Arabidopsis*, and other genes were extracted from the leaves. The reaction mixture of 20 μL was composed of 1 μL of cDNA, 0.4 μL of each primer, 10 μL of SYBR^®^Premix Ex Taq™ (TaKaRa, Inc., Tokyo, Japan) and 8.2 μL of ddH_2_O. The PCR program consisted of an initial step of 95 °C for 30 s, which was followed by 40 cycles of 95 °C for 5 s and 60 °C for 30 s and then a dissociation stage of 95 °C for 10 s, 65 °C for 5 s and 95 °C for 5 s. Each gene was assessed in three biological replicates, and the relative expression levels were calculated using the 2^−ΔΔCt^ method.

### 4.7. Vector Construction and Arabidopsis Transformation

The full-length CDSs of *PmAGL6-1* and *PmAGL6-2* were cloned into the *pCAMBIA1304* vector under the control of the *CaMV35S* promoter. These plasmids were then transformed into an *Agrobacterium tumefaciens* strain for inflorescence infection, which was used to transform *Arabidopsis.* Mature T0 seeds were collected, disinfected and then sown on 1/2 MS medium that contained 30 mg/mL hygromycin to screen the transformants. The resistant plants grew normally; they were removed and transplanted into small flowerpots and grown at 21 °C under long-day conditions. Screening was repeated according to the above methods until T2 plants were obtained.

### 4.8. Yeast Two-Hybrid Assay

The Matchmaker Gold Yeast Two-Hybrid System (Clontech, Mountain View, CA, USA) was used for Y2H assays. The full-length CDSs of *PmAGL6-1* and *PmAGL6-2* were recombined into the pGBKT7 vector to express the bait proteins. The full-length CDSs of *PmAGL6-1, PmAGL6-2, PmAP1, PmFUL1, PmFUL2, PmAP3, PmAP3-2, PmPI, PmAG, PmSEP1, PmSEP2, PmSEP3* and *PmSEP4* were recombined into pGADT7. These two types of fusion vectors were cotransformed into Y2Hgold cells. The transformed Y2H Gold strain was cultured on SD/-Leu/-Trp and SD/-Trp/-Leu/-Ade/-His media with aureobasidin A (AbA) and X-α-Gal and cultured at 28 °C for three days.

### 4.9. Bimolecular Fluorescence Complementation Assay

The full-length CDSs of *PmAGL6-1* and *PmAGL6-2* without termination codons were subcloned into the pSPYCE vector, and the full-length CDSs of *PmAGL6-1, PmAGL6-2, PmAP1, PmFUL1, PmFUL2, PmAP3, PmAP3-2, PmPI, PmAG, PmSEP1, PmSEP2, PmSEP3* and *PmSEP4* without termination codons were subcloned into the pSPYNE vector.

These fusion vectors were transfected into *A. tumefaciens* strain GV3101. *Agrobacterium* cells positive for the two types of fusion vectors were coinfiltrated into *N. benthamiana* leaves, which was followed by culturing in darkness. After 2–3 days, YFP fluorescence was observed using a confocal laser microscope.

## 5. Conclusions

The full-length CDS sequences of *PmAGL6-1* and *PmAGL6-2* were first cloned from the cDNA of *P. mume* flower buds. *PmAGL6-1* and *PmAGL6-2* are not expressed in vegetative organs (leaves, roots and stems) but are highly expressed in fruits, and there are differences in expression patterns in floral organs. *PmAGL6-1* is expressed in sepals, petals, stamens and pistils and is highly expressed in sepals and petals, while *PmAGL6-2* is only expressed in sepals and pistils and is highly expressed in sepals. *PmAGL6-1* and *PmAGL6-2* increased continuously with the flower bud differentiation, and the expression of *PmAGL6-1* reached the highest level at the ovule development stage (S6) and *PmAGL6-2* reached the highest level at the pistil initiation (S5) stage, and then the expression of these two genes was downregulated. The expression patterns of *PmAGL6-1* and *PmAGL6-2* indicated that they may be involved in the development of floral organs and fruits. According to the bioinformatics analysis, expression patterns, protein–protein interaction patterns and phenotypic changes in transgenic *Arabidopsis*, *PmAGL6-1* and *PmAGL6-2* play important roles in promoting flowering and flower organ development. In addition, *PmAGL6-1* and *PmAGL6-2* may also participate in the development of floral meristems and fruits.

## Figures and Tables

**Table 1:** Primers for qPCR.

Primers	Primers Sequence
q*PmAGL6-1*-F	TCTCGGACCTCTGAATGTGA
q*PmAGL6-1*-R	TTCTGTCTCCAGCTTGACC
q*PmAGL6-2*-F	ACCGTCAATGTTGCTACTCC
q*PmAGL6-2*-R	TCACCAGTTCTTTCAGGCGAA
qPP2A-F	ATATAGCTGCTCAGTTCAACC
qPP2A-R	AAAAACAGTCACCACATTCTT
qActin-F	TCTCTATGCCAGTGGTCGTA
qActin-R	CCTCAGGACAACGGAATC
qAP1-F	CAGATCAAGGAGAGGGAA
qAP1-R	TTGATACAGACCACCCAT
qSOC-F	CTCCAATATGCAAGATACCA
qSOC-R	TATGCCTTCTCCCAAGAGTT
qLFY-F	CCCAAGAAGGGTTATCTGA
qLFY-R	AAACGGATGCTCCCTCTG
qCO-F	CCATTAACCATAACGCATAC
qCO-R	GTCAGGTTGTTGCTCTACT
qFT-F	TGGAACAACCTTTGGCAATG
qFT-R	GTCTTCTTCCTCCGCAGC
qCYC-F	AAGGCTTTGAGTTTCCTGAGGTG
qCYC-R	TGAGCAGCCAGTCTAACGTTTTAC
qFLC-F	CGAAGCTGATAATATGGAGATGT
qFLC-R	AGATATACAACGTGCACCCTTCA
PmAGL6-1F	ATGGGGAGAAGGAAAGTGGTG
PmAGL6-1R	TCAAAGAACCCATCCCTG
PmAGL6-2F	ATGGGGAGAAGGAAAGTGGTG
PmAGL6-2R	TTAAAGAAGCCAACCCCGC

**Table 2:** Days needed for flowering and number of rosette leaves.

Transgenic Line/WT	Days Needed for Flowering	Number of Rosette Leaves	Transgenic Line/WT	Days Needed for Flowering	Number of Rosette Leaves
WT	42.20 ± 1.66a	10.30 ± 1.37a	B1	31.80 ± 0.51bc	7.50 ± 0.40c
A1	26.80 ± 0.80d	7.40 ± 0.68c	B2	33.40 ± 1.27b	7.90 ± 1.38bc
A2	30.20 ± 0.74c	7.80 ± 0.49bc	B3	31.60 ± 0.52bc	8.20 ± 0.36bc
A3	32.10 ± 1.74bc	8.40 ± 1.68bc	B4	30.40 ± 0.69bc	7.20 ± 0.73bc
A4	29.20 ± 0.75c	70.60 ± 0.98bc	B5	33.01 ± 1.17b	7.40 ± 0.48c

Significant differences are identified by SPSS with Duncan’s test (*p* < 0.05) and are represented by different letters above the error bars.

## Data Availability

All data in this study can be found in the manuscript.
